# Dengue seroprevalence, seroconversion and risk factors in Dhaka, Bangladesh

**DOI:** 10.1371/journal.pntd.0005475

**Published:** 2017-03-23

**Authors:** Parnali Dhar-Chowdhury, Kishor Kumar Paul, C. Emdad Haque, Shakhawat Hossain, L. Robbin Lindsay, Antonia Dibernardo, W. Abdullah Brooks, Michael A. Drebot

**Affiliations:** 1 Zoonotic Diseases and Special Pathogens, Public Health Agency of Canada, National Microbiology Laboratory, Winnipeg, Manitoba, Canada; 2 Natural Resources Institute, University of Manitoba, Winnipeg, Manitoba, Canada; 3 Emerging Infections, International Centre for Diarrheal Disease Research, Bangladesh, Dhaka, Bangladesh; 4 Department of Mathematics and Statistics, University of Winnipeg, Winnipeg, Manitoba, Canada; 5 Center for Global Health, Johns Hopkins University, Baltimore, Maryland, United States of America; Naval Medical Research Center, UNITED STATES

## Abstract

**Background:**

Dengue virus (DENV) activity has been reported in Dhaka, Bangladesh since the early 1960s with the greatest burden of dengue fever and dengue hemorrhagic fever cases observed in 2000. Since this time, the intensity of dengue activity has varied from year to year, and its determining factors remained relatively unknown. In light of such gaps in knowledge, the main objectives of this study were to determine the magnitude of seroprevalence and seroconversion among the surveyed population, and establish the individual/household level risk factors for the presence of DENV antibodies among all age groups of target populations in the city of Dhaka.

**Methodology/Principal findings:**

Considering the lack of fine scale investigations on the factors driving dengue activity in Bangladesh, a prospective cohort study involving serological surveys was undertaken with participant interviews and blood donation across the city of Dhaka in 2012. Study participants were recruited from 12 of 90 wards and blood samples were collected during both the pre-monsoon (n = 1125) and post-monsoon (n = 600) seasons of 2012. The findings revealed that the seroprevalence in all pre-monsoon samples was 80.0% (900/1125) while the seropositivity in the pre-monsoon samples that had paired post-monsoon samples was 83.3% (503/600). Of the 97 paired samples that were negative at the pre-monsoon time point, 56 were positive at the post-monsoon time point. This resulted in a seroprevalence of 93.2% (559/600) among individuals tested during the post-monsoon period. Seroprevalence trended higher with age with children exhibiting a lower seropositivity as compared to adults. Results from this study also indicated that DENV strains were the only flaviviruses circulating in Dhaka in 2012. A multivariate analysis revealed that age, possession of indoor potted plants, and types of mosquito control measures were significant factors associated with DENV seroprevalence; while attendance in public/mass gatherings, and use of mosquito control measures were significantly associated with DENV seroconversion after adjusting for all other variables.

**Conclusions/Significance:**

Our study suggests that there is a high level of endemic dengue virus circulation in the city of Dhaka which has resulted in significant DENV seroprevalence among its residents. Seropositivity increased with age, however, a substantial proportion of children are at risk for DENV infections. Our serological analysis also documents considerable DENV seroconversion among study participants which indicates that a large proportion of the population in the city of Dhaka were newly exposed to DENV during the study period (pre-and post-monsoon 2012). High levels of seroconversion suggest that there was an intense circulation of DENV in 2012 and this may have resulted in a significant risk for viral associated illness. Findings of our study further indicated that home-based interventions, such as removing indoor potted plants and increased bed net use, in addition to vector control measures in public parks, would reduce exposure to DENV and further decrease risk of viral associated disease.

## Author summary

Similar to many other tropical regions of the world, dengue is a major public health problem in Bangladesh where *Aedes aegypti* mosquitoes are the main vector. Through this serological survey, we present data on the magnitude (measured in proportions) of seroprevalence and seroconversion within 12 selected wards in the city of Dhaka, Bangladesh. In 2012 the observed dengue seroprevalence was 93% among individuals tested during post-monsoon with a seroconversion rate between pre- and post-monsoon periods of 57.7%. This finding suggests that dengue virus (DENV) circulated in the city during the observation period. Past exposure to dengue virus was highly associated with age, possession of indoor potted plants, types of mosquito control measures used, and human movement and attendance in mass gatherings, while the extent of dengue seroprevalence were not associated with the socioeconomic status of the study participants. Our findings suggest that household utilities and water management or storage practices and recognition and elimination of mosquito development sites and participation in mass gatherings are important factors that affect exposure to dengue. Intervention strategies should therefore target these factors for effective prevention and control of dengue infection.

## Introduction

The spread of dengue virus (DENV)–a viral pathogen transmitted by mosquitoes, primarily *Aedes aegypti* and *Ae*. *albopictus–*has been unprecedented in recent decades. At the present time, 390 million people are exposed to DENV each year resulting in 96 million annual cases of viral associated disease globally [1] while approximately 3 billion people living in the tropical and subtropical regions are at risk of infection [2–6]. According to estimates from the World Health Organization (WHO), approximately 500,000 people develop severe disease each year, and among them, about 1,250 (2.5%) die [7]. The reasons for increased incidence and rapid geographic spread of dengue are not fully understood; however, numerous ecological, biological, social, economic, and cultural factors could be responsible. Due to the fact that the most advanced dengue vaccine candidate has hitherto exhibited only intermediate efficacy [8] despite considerable investment and research, the current dengue prevention and epidemic response primarily relies upon vector control measures designed to reduce vector abundance. Effective vector control requires a clear understanding of the drivers and/or determining factors which drive DENV transmission. It is therefore important to determine the epidemiological, socioeconomic and ecological factors and the dynamics of exposure to DENV infection, risk and epidemic potential.

Among numerous factors, globalization of the world economy, increases in international travel, breakdowns in public health infrastructure, ineffective vector control programs and enhanced climate variability have been cited for their potential roles in resurgence of DENV [1,9–10]. The underlying drivers of DENV activity operate at various spatial scales, and among factors at the regional/local scale, uncontrolled urbanization [11], human population density and clustering of households with DENV activity seem to play a prominent role. At the finer scale of neighborhoods and households, the absence of public utility services including reliable sources of potable water, effective sewer systems, and appropriate waste disposal appears to contribute significantly to the problem [12–15]. The complexity of the factors influencing the dynamics of patterns of DENV transmission are reflected in the heterogeneity of dengue incidence in time and space and this heterogeneity needs to be considered when attempting to understand or predict patterns of disease incidence.

Although the first recognized outbreak of dengue in the city of Dhaka, the capital of Bangladesh, was recorded in 1964 [16], followed by sporadic cases of dengue fever (DF) during 1977–78 and 1996–1997 [17], the extent of dengue prevalence in Bangladesh is poorly documented. It is also possible that sporadic or ongoing transmission occurred during the 1964 to 1978 and 1978 to 1997 time frames, but went undetected, due to under-reporting. The first identified epidemic of DF and dengue hemorrhagic fever (DHF) in Bangladesh, took place during the monsoon season of 2000, and resulted in 5,521 officially reported cases with 93 fatalities [17–18]. Based on molecular diagnostic testing of persons with acute disease, all four dengue serotypes have been found in circulation in recent years [19–21]. From 2000–2009, 91.0% of all reported dengue cases were from Dhaka making it the most endemic urban area of the country [22]. Cases of DF were clustered during the time periods: 2000–2002, 2003–2005, and 2006–2009, and Dhaka was the locality with ‘the most likely cluster for DF in all three periods’ [18]. Since 2009, the number of reported dengue cases declined in Bangladesh [23]; however, as discussed by Sharmin et al. [22], this was likely an artifact of changes in reporting criteria (i.e., laboratory confirmation was required in order to confirm cases instead of using a clinical diagnosis). As a result, the ‘tip of the iceberg’ analogy of dengue reporting is likely in play in Bangladesh, as it has been well-documented that passive surveillance involving case notifications does not accurately reflect the burden of dengue in such a geographical location [24].

The local drivers of dengue activity in Bangladesh are poorly defined. For example, Mahmood and Mahmood [25] suggested that several macro-level risk factors including overpopulation, uncontrolled urbanization, and poor waste management play prominent roles in the emergence of dengue in Bangladesh. Furthermore, piped water supply, drainage and adequate sewage disposal are unevenly distributed throughout the city, all of which can have an effect on dengue transmission [26]. At the present time, detailed studies on the factors operating at the household level that impact dengue transmission dynamics are generally lacking in Bangladesh.

Given the absence of human surveillance system for dengue and a lack of fine scale investigations on the factors driving dengue activity in this country, a prospective cohort study involving serological surveys was undertaken with participant interviews and serological sampling across the city of Dhaka in 2012. Because dengue incidence in Bangladesh is highly monsoon dependent [10], two separate household serosurveys were conducted—one pre-monsoon (to estimate seroprevalence) and one post-monsoon (to determine the extent of seroconversion). The target population included all household members including children less than 12 years of age. Potential risk factors for dengue exposure were solicited from data collected during structured participant interviews. To the best of our knowledge, this study is the first population serosurvey on dengue in Bangladesh, with the objective i) to determine the magnitude of dengue seroprevalence and seroconversion, and ii) simultaneously to determine the individual/household level risk factors among all age groups for dengue transmission in various socioeconomic status zones of the city of Dhaka. It was anticipated that the outcomes of this investigation would inform epidemic risk management authorities and assist in the development of effective intervention tools and strategies.

## Methods

### Study site and population

The city of Dhaka is ranked as the 9th largest city in the world [27]; it is also among the most densely populated cities. Considering that the city of Dhaka has experienced the most intense dengue activity in Bangladesh in recent years [18] and it has been cited as the most endemic area of the country [22], Dhaka City Corporation [(DCC) location: 23.77° N; longitude, 90.38° E)] was chosen as the study area for the present investigation. Dhaka experiences a hot, wet and humid tropical climate with a distinct monsoonal season. The annual average temperature is 28°C and annual average rainfall of 2002 mm [28]. The pre-monsoon season is very hot with an average maximum of 36.7°C, a very high rate of evaporation and erratic but occasional heavy rainfall from March to June. The post-monsoon season is short-lived and characterized by decreased rainfall and gradual lowering of night time minimum temperatures.

More than one-third of the population of this city is poor and lives in dense squatter settlements, and their livelihood usually relies on daily wage earnings [29]. The city has the natural and social conditions which provide ample larval development sites for mosquito populations, particularly during the monsoon, rainy seasons. *Aedes aegypti*, as the primary vector, occupies the overwhelming majority of the wards (administrative units) in Dhaka [30], while the secondary vector–*Aedes albopictus*–occupies only a few wards in and around the fringe areas [31–32].

### Population sampling

The primary sampling units of this study were individual households (HHs). A multi-stage, stratified sampling design was used for this investigation. The 90 administrative wards (according to the previous administrative system) of the city were categorized by socio-economic status (SES) (i.e. high, medium, and low). The criteria for classifying the wards, following a Delphi method, into three SESs are described elsewhere [30]. A probability proportional sampling (PPS) method [33] was employed to select sample wards from each stratum, which resulted in 2 wards from high, 5 wards from medium, and 5 wards from low SES. Finally, a spatial randomization procedure was followed where 100m X 100m grid cells were superimposed on each ward, and a total of 100 grid cells were randomly selected. From each ward, 100 households were selected as sampling units for the surveys, resulting in a total of 1200 target households (i.e., 200 from high, 500 from medium, and 500 from low SES wards). Initially, the project was designed to examine i) the seroprevalence of DENV antibodies in the target populations and ii) the prevalence, abundance and distribution of DENV vector mosquitoes. As a result, no specific clinical information was used for recruitment.

### Study design

For the purpose of this study, *seroprevalence* was defined as the percentage of total individuals in a population who tested positive for the presence of IgG antibodies to DENV. Individuals identified as *seroconverted* were found to be negative for dengue antibody during the pre-monsoon period but who became positive for dengue IgM or IgG based on the serological analysis of their post–monsoon sample. The extent of seroconversion refers to the percentage of new cases as well as new exposures identified between the pre- and post-monsoon period.

Two household serosurveys were conducted in 2012 in order to assess the seroprevalence and seroincidence of DENV. The first serosurvey was completed during the pre-monsoon season of 2012 (June and July). At this time, information on demographic, socio-economic and other relevant characteristics was collected from the study participants using a structured socioeconomic questionnaire. The inclusion criteria for household members in our study were: all household members shared one roof, all meals, and common living space. Participants were *a priori* required to give informed consent. Special attention was given to age, sex, place of residence, socioeconomic status, water storage and supply, and migration and intra-city mobility variables. The 2012 pre-monsoon serosurvey served as a baseline for the determination of DENV seroprevalence and involved the testing of 1125 individuals.

The follow-up serosurvey was carried out during the 2012 post- monsoon (November) and households that participated in the baseline serosurvey were revisited. At the time of the follow-up study, 600 of the 1125 (53.2%) individuals still resided in the previously recorded locations and were willing to provide another blood sample. Thus, these 600 study participants (age range: 1–77 years of age) from 390 households who provided paired serum samples comprised the sample set for determining the extent of seroconversion between the 2012 pre- and post-monsoon time frames.

### Blood sample collection

Participation in the serosurvey was on a voluntary basis, and in case of children (1–11 years of age) consent of both individuals and parents (whenever possible) was obtained. Blood samples were collected by personnel from the International Centre for Diarrhoeal Disease Research, Bangladesh (ICDDR,B) in Dhaka. The ethical guidelines of the Bangladesh Medical Research Council were followed for the collection of 5-mL of intravenous blood from each participant. The serum samples were separated by centrifugation and stored at -20°C in ICDDR,B laboratories; a unique identification number was assigned to each participant to maintain anonymity. All serum samples were sent to the National Microbiology Laboratory (NML) in Winnipeg, Canada where they were stored at -80° C until tested.

### Serologic analysis

All sera collected during the 2012 pre-monsoon (n = 1125) and post-monsoon (n = 600) were tested for antibodies to DENV using commercial IgM and IgG ELISA kits. Because flavivirus cross reactivity is a concern in ELISAs, and other flaviviruses have been sporadically reported in Bangladesh [77,78], a subset of 100 IgG positive sera from 2012 pre-monsoon and 50 IgG positive sera from post-monsoon were randomly selected and tested for Japanese encephalitis virus (JEV), West Nile Virus (WNV) and DENV-neutralizing antibodies by plaque reduction neutralization tests (PRNT) as previously described [34].

### Enzyme-linked Immunosorbant Assay (ELISA)

Serum samples collected during the pre- and post-monsoon were tested for dengue IgG and IgM antibodies by ELISA. Sera were tested at a 1:100 dilution using the DENV IgG Dx Select and DENV IgM Capture Dx Select kits (Focus Diagnostics Inc., Cypress, CA, USA) according to the manufacturer’s instructions. In order to mitigate potential false positive reactions due to heterophilic IgM antibody activity, samples that were initially positive in the IgM ELISA were re-tested using the background subtraction procedure as detailed in the kit instructions.

### Plaque reduction neutralization test (PRNT)

A total of 150 IgG-seropositive samples from across age groups (135 adults and 15 children under 12 years of age) were randomly selected and tested for DENV-neutralizing antibodies using a PRNT assay [34]. A known amount of DEN 2 virus [i.e., 100 plaque forming units (PFU)] was mixed with various dilutions of test sera and incubated for 90 minutes at 37°C. Following this, 100 μl of the virus-serum mixture was added to fully confluent Vero cell monolayers (in 6 well tissue culture plates) and incubated for one hour at 37°C with gentle rocking every fifteen minutes. Following incubation, 3 mls of an agar overlay was applied to each well and the plates were incubated at 37°C, 5% CO_2_ for 72–120 hours depending on the virus. A second agar overlay containing the vital dye neutral red was added and the presence/absence of plaques was noted after an additional 18 hours of incubation. By determining the final dilution of serum that lead to a 90% reduction in plaque formation (PRNT_90_), an end-point titre for virus-specific neutralization activity was calculated. For screening purposes samples with PRNT_90_ titers ≥ 20 were considered to have neutralizing antibody against DENV or other flaviviruses. End point titres of 4-fold or greater difference between dengue and WNV or JEV were used to identify the specific flavivirus antibodies in the selected serum samples.

### Data analysis

Among the 1,200 selected households for baseline seroprevalence as well as the follow-up survey, data obtained from the participants were analyzed. This study initially targeted one participant from each household; however, multiple serum samples were sometimes collected from each household when more than one household member was willing to participate. Two outcome variables (i.e., seroprevalence and seroconversion/ recent DENV infection) for dengue infection were defined based on serological testing. Univariate and multivariate analyses of potential socioeconomic and demographic risk factors for DENV infection were performed using SPSS (version 22.0; SPSS Inc., Chicago, IL). Age-and gender-specific antibody prevalence, with 95% confidence intervals (CI) were calculated. The χ ^2^ test was used to evaluate the strength of the association between outcome variables and the risk factors, as described in Dhar-Chowdhury et al. [30], and it was measured by prevalence odds ratios (OR), with 95% CIs. A p-value of <0.05 was considered statistically significant. To control for potential confounders and to assess the effect of each risk factor a logistic regression was constructed, and the corresponding ORs with 95% CIs were also estimated. Variables that were related to DENV IgG seroprevalence and seroconversion in the bivariate analysis (p-value<0.25) were included in the logistic regression models. A Receiver Operating Characteristic (ROC) curve was drawn to assess the goodness-of-fit of our logistic regression model. All analyses were performed using statistical software SAS 9.1.3 (SAS Institute. Cary, NC, USA).

### Ethical considerations

The research was approved by the Bangladesh Medical Research Council (Bangladesh) and the Joint Faculty Research Ethics Board of the University of Manitoba (Canada). The purpose and objectives were explained to the head of each household and her/his informed consent was sought to collect demographic, socioeconomic, household infrastructure and ecological information [30]. In Bangladesh, many respondents are illiterate and are unwilling to provide written consent due to fear of forgery or deception. In this regard, both Bangladeshi and Canadian ethics Boards, considering the socio-cultural contexts, approved oral consent procurement, with witnesses and their signatures. Written (with signature) or verbal consent (recorded in tape recorders with consent) was also obtained from each household member who donated blood samples; in case of children, consent from parent(s) was obtained.

## Results

### Outcomes of study population

A total of 1125 serum samples [1003 (89.2%) from adults and 122(10.8%) from children under 12] were collected during the 2012 pre-monsoon from residents of 12 wards of the Dhaka City Corporation from a total of 635 households. The descriptive analysis was performed for each variable of our study population (Table 1). The age of survey participants ranged from 1 to 77 years with a mean age of 31.9 years (95% CI: 29.0–31.0). The male to female ratio of the subjects was 1:1.3 and more than half of the participants were housewives (34%) or students (19.6%).

**Table 1:** Demographic and socioeconomic background characteristics of serosurvey (2012 pre-monsoon) participants (N = 1125).

Population characteristics	Count	%
**Age (years)**		
< 12	122	10.8
12–22	251	22.3
23–33	320	28.4
34–44	225	20.0
45–55	139	12.4
56–66	60	5.3
67–77	8	0.7
**Sex**		
Male	487	43.3
Female	638	56.7
**Income (Bangladeshi Taka)**		
0–14,999	441	39.2
15000–24,999	226	20.2
≥25, 000	453	40.4
Non-response	5	0.4
**Education**		
No Education	122	10.8
Primary	352	31.3
Secondary	293	26.0
Higher Secondary	140	12.4
Undergraduate	135	12.0
Graduate	83	7.4
**Occupation**		
Service/professional	44	3.9
Business	124	11.0
Labor/menial workers	135	12.0
Student	221	19.6
Housewife	383	34.0
Others	116	10.3
Non-response	102	9.1

### DENV-specific IgG and IgM results

**Fig 1:**
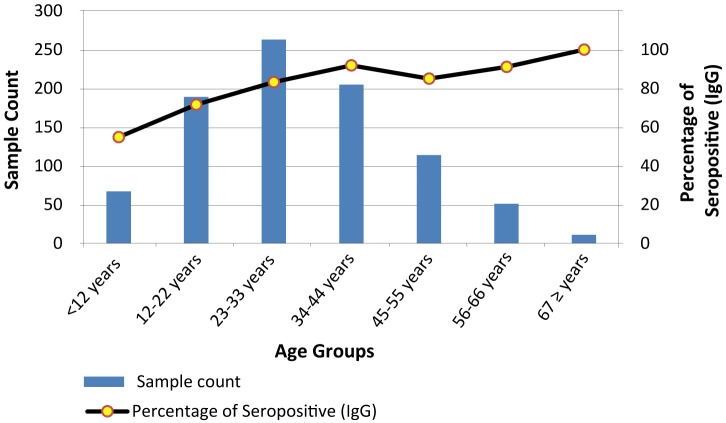
Number of counts (sample) and percentage of seropositive (IgG) cases by age groups (all ages), 2012 pre-monsoon serosurvey.

During pre-monsoon survey, the overall DENV IgG seroprevalence was 80% (900 / 1125). As shown in Fig 1, among adults, 83.1% (833 of 1003) were positive for IgG while 54.9% (67 of 122) of children had DENV IgG antibodies. Overall, 22 of 1003 (2.2%) adults and 1 of 122 (0.8%) children had IgM antibodies which suggests that recent infections with DENV were occurring. In general, adults who were 34–44 years of age and older had a high (92%) seroprevalence of DENV antibodies and seroprevalence generally increased with age (Fig 1; S1 Table).

During the post-monsoon survey, paired sera were collected from a subset of 600 individuals (53.3% of the original 1125 participants) residing in 390 households. The seroprevalence for these 600 individuals increased from 83.8% (503 / 600, pre-monsoon) to 93% (559/600) during the monsoon period indicating a significantly higher magnitude of DENV circulation and seroconversion during this time frame (Table 2).

**Table 2:** Proportion of individuals in which antibodies to dengue were detected in paired blood samples collected during the pre- and post-monsoon seasons in 2012, Dhaka, Bangladesh.

Serological status	Pre-Monsoon	Post-Monsoon
	n (%)	n (%)
Seropositive individuals	503 (83.8)	559 (93.2)
Seronegative individuals	97 (16.2)	41 (6.8)
All individuals	600 (100)	600 (100)

Consistent with this pre- to post-monsoon increase in seroprevalence, 57.7% (56 of 97) of individuals (600 participants with paired sera) who were seronegative during the pre-monsoon survey had seroconverted to either IgG (n = 47 of 56) or IgM antibodies (n = 4 of 56) or both immunoglobulins (n = 5 of 56). Overall, there was a higher number of seroconversions among female (60.7% CI: 0.50–0.71) participants than males (39.3% CI: 0.29–0.50) and among adults 75% (42 /56) than children 25% (14/56). For adults, 59% (42/71) seroconverted between the pre- and post-monsoon periods. Among children (<12 years of age), the extent of seroconversion was somewhat lower at 53.8% (14/26). For children 6 years of age or more, seroconversion was determined to be 56.2% (9/16); while 50% (5/10) of children less than 6 years old seroconverted.

**Fig 2:**
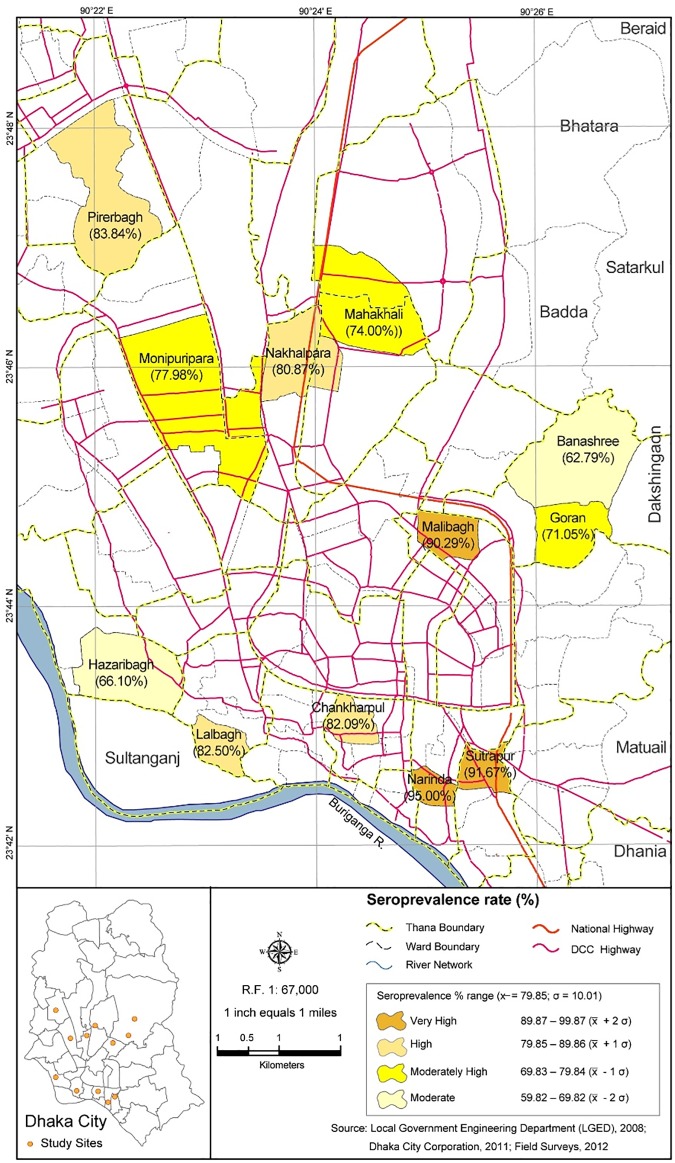
Distribution and characteristics of seroprevalence by city-wards during 2012 pre-monsoon serosurvey in Dhaka, Bangladesh.

Fig 2 illustrates the geographical distribution of seroprevalence by ward, and the proportion of seroprevalence varied among the wards from moderate/moderate high (59.8–79.8%) to high/very high (79.8–99.9%). The very high degree of seroprevalence was observed in the older settlements, located in the southern and central zones of the city whereas moderate seroprevalence levels prevailed in the fringe areas.

### DENV-neutralizing antibody profiles

There was a strong positive correlation between the results obtained with the IgG ELISAs and the PRNT assay. Of the 150 IgG ELISA-positive samples, 96% and 94% of the pre- and post-monsoon samples, respectively, had neutralizing titers (≥ 1:20) to DENV-2. Although a number of samples generated positive titres (1:20 dilution) for all three flaviviruses, endpoint titration performed on these samples were negative for JEV and WNV when the 4-fold difference in virus-specific titres was used to differentiate the identity of the infecting virus. At the lowest screening dilution factor (i.e., 1:20) cross-reactivity in PRNTs can be observed in flavivirus infections because antibodies elicited against conserved epitopes on the immunogenic envelope protein may cross-react with other flaviviral antigens. Low neutralizing titres to other flaviviruses may therefore occur, leading to the false-positive results on the screening assays. The PRNT results confirmed the absence of JEV and WNV antibodies. Hence, the seroprevalence identified in this study can be regarded as resulting from ‘confirmed dengue infections’.

### Demographic and socioeconomic risk factors

**Fig 3:**
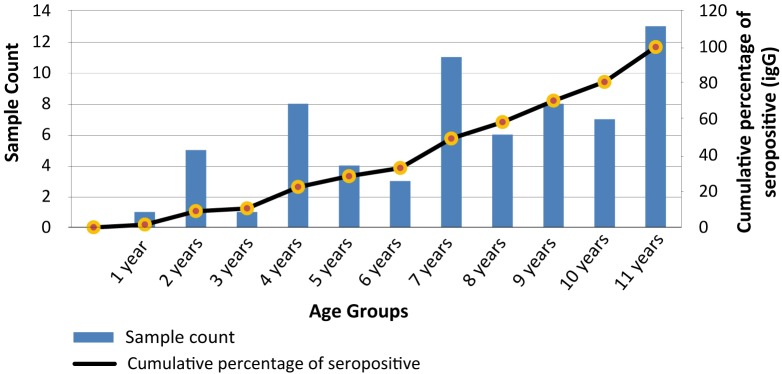
Number of counts (sample) and cumulative percentage of seropositive (IgG) cases among the children (≤12 years) by year, 2012 pre-monsoon serosurvey.

The univariate analyses [dependent variable was serological status (i.e., either positive or negative)] confirmed significant association between IgG seroprevalence and all different age groups (CI: 2.70–5.90; p-value <0.0001) (Table 3). From Fig 3 (S2 Table), we find that among children (<12 years of age), the IgG prevalence increased with age; by the age of 9 years, 77% (94 of 122) children were IgG seropositive. There was no significant difference in seroprevalence by sex (p-value = 0.372). In addition to age, number of indoor potted plants (CI: 0.34–0.77; p-value = 0.0011), types of mosquito control measures (CI: 0.93–2.93; p-value = 0.016), and febrile illness in any family members during the past six months (CI: 1.00–1.81; p-value = 0.0441) showed statistically significant association with seroprevalence (Table 3).

**Table 3:** Univariate association between seroprevalence (IgG) of dengue and selected explanatory variables during the 2012 pre-monsoon serosurvey.

Variable	Seropositive	Seronegative	OR	p
	n (%)	n (%)	(95% CI)	
**Age (years)**				
< 12 years (children)	67 (7.4)	55 (24.3)	1	<0.0001^★^
12–77 years (adults)	833 (92.6)	170 (75.7)	4.00 (2.70–5.90)	
**Household income**				
< 14,9999	341 (38.0)	100 (44.8)	1	0.0827
15000–24,999	179 (20.0)	47 (21.1)	1.10 (0.76–1.65)	
≥ 25,000	377 (42.0)	76 (34.1)	1.46 (1.04–2.03)	
**Sex**				
Female	504 (56)	134 (59.3)	1	0.3720
Male	395 (44)	92(40.7)	1.14 (0.85–1.54)	
**Number of uncovered water tank outdoor**				
1	475 (52.8)	126 (55.5)	1	0.506
≥ 1	423 (47.2)	101 (44.5)	1.10 (0.82–1.48)	
**Number of indoor potted plants**				
≥ 1	218 (24.3)	32 (14.2)	1	0.0011^★^
None	681 (75.7)	194 (85.8)	0.52 (0.34–0.77)	
**Attendance in public gatherings**				
No attendance	249 (27.7)	76 (33.6)	1	0.256
Schools	211 (23.4)	54 (23.9)	1.19 (0.80–1.77)	
Mosque/religious institutions	145 (16.1)	29 (12.8)	1.53 (0.95–2.45)	
Entertainment places/ festivities	294 (32.8)	67 (29.7)	1.34 (0.93–1.94)	
**Types of mosquito control measure**				
Bed net	243 (33.2)	62 (31.8)	1	0.016^★^
Mosquito coil	323 (44.1)	105 (53.8)	0.79 (0.55–1.12)	
Spray and other	166 (22.7)	28 (14.4)	1.51 (0.93–2.93)	
**Any family member suffered from febrile illness within last 6 months**				
Yes	446 (49.6)	129 (57.1)	1	0.0441^★^
No	453 (50.4)	97 (42.9)	1.35 (1.00–1.81)	

^★^Significant at p = 0.05 level

Results of the univariate analyses depicted in Table 4 revealed that seroconversion (i.e., seroconversion/ person/ season) was significantly associated with attendance in mass/public gatherings (p value = 0.032) and type of mosquito control measures (p value = 0.029). There was no statistically significant difference in the seroconversion by sex, age, occupation, education, income, number of water vessels indoors, number of indoor potted plants, vegetation/trees nearby and family members suffered from febrile illness.

**Table 4:** Univariate association between seroconversion of dengue and selected individual variables during follow-up survey (post-monsoon 2012).

Variables	Seropositive n (%)	Seronegative n (%)	OR	p-value
Mean Age (year)				
Age (year)				
< 12 years (children)	14 (25.0)	13 (31.7)	1	
12–77 years (adults)	42 (75.0)	28 (68.3)	1.5 (0.61–3.71)	0.38
**Sex**				
Male	22 (39.3)	17(41.5)	1	
Female	34 (60.7)	24 (58.5)	1.10 (0.48–2.49)	0.834
**Income in household**				
0–14,999	16 (28.6)	9 (22.0)	1	
15,000–24,999	24 (42.8)	11 (26.8)	1.23 (0.42–3.63)	
> = 25,000	16 (28.6)	21(51.2)	0.43 (0.15–1.22)	0.076
**Education**				
Less than Secondary	29 (51.8)	26 (63.4)	1	
Secondary and Above	27 (48.2)	15 (36.6)	1.61 (0.71–3.68)	0.254
**Occupation**				
Services (Professional, business, labor etc.)	9 (16.1)	10 (24.4)	1	
Non-wage earning (Student, Housewife, etc.)	42 (75.0)	27 (65.9)	02.52 (1.20–5.28)	0.294
No Response	5 (8.9)	4 (9.7)		
**Attendance in public/mass gatherings**				
Garden/Park	21 (37.5)	7(17.1)	1	
Others (Schools, Religious Institutions etc.)	21 (37.5)	22 (53.7)	0.32 (0.11–0.90)	0.032*
No Response	14 (25.0)	12 (29.2)		
**Number of water vessels inside household**				
None	14 (25.0)	15 (36.6)	1	
≥1	42 (75.0)	26 (63.4)	0.58 (0.24–1.39)	0.221
**Number of indoor potted plants**				
None	45 (80.4)	35(85.4)	1	
≥1	11 (19.6)	6 (14.6)	0.70 (0.24–2.08)	0.522
**Vegetation/tree nearby**				
Yes	38 (67.9)	23 (56.1)	1	
No	18 (32.1)	18 (43.9)	0.61 (0.26–1.39)	0.238
**Types of mosquito control measures**				
Bed nets	17 (30.4)	24 (58.5)	1	
Mosquito coils	25 (44.6)	11 (26.8)	3.21 (1.25–8.24)	
Sprays and others	6 (10.7)	2 (4.9)	4.24 (0.76–23.57)	0.029*
No Response	8 (14.3)	4 (9.8)		
**Any family member suffered from febrile illness during last 6 months**				
Yes	28 (50.0)	23(56.1)	1	
No	28 (50.0)	18(43.9)	1.28 (0.57–2.87)	0.552

*Significant variables at p = 0.05 level

### Multivariate analyses

In order to determine whether socioeconomic (i.e., income, education and occupation), demographic (age, sex), behavioral (i.e., attendance in public gatherings, types of mosquito control measures used), utensils (i.e., water vessels outdoors, number of potted plants), and epidemiological (i.e., febrile illness among any family members in past six months) factors were associated with seroprevalence, after adjusting the explanatory variables, a multivariable logistical regression model with stepwise selection was applied. The model outputs shown in Table 5 revealed that age (χ^2^ = 18.4, df = 2, p-value <0.0001), number of indoor potted plants (χ^2^ = 8.3, df = 1, p-value = 0.004), and type of mosquioto control measures used (χ^2^ = 6.1, df = 2, p-value = 0.048) were significantly associated with seroprevalence after adjusting all other variables.

**Table 5:** Estimated odds ratios (OR) for the multilevel analysis of the association between seroprevalence and selected explanatory variables from univariate analysis of 2012 pre-monsoon serosurveys data.

Variable	Estimates (St. error)	OR (95% CI)	p-value
**Age**			0.05
Intercept	0.59 (0.30)		
0–11		1	<0.0001^★^
12–44	1.42 (0.23)	4.13 (2.65–6.42)	
45 and over	1.78 (0.30)	5.90 (3.26–10.68)	
**No. of indoor potted plant**			
One and more		1	0.004^★^
None	-0.63 (0.22)	0.53 (0.34–0.81)	
**Type of mosquito control Measure**			
Bed nets		1	0.048^★^
Mosquito coils vs Bed nets	-0.22 (0.19)	0.80 (0.56–1.16)	
Sprays or others vs Bed nets	0.36 (0.26)	1.44 (0.87–2.39)	

OR = Odds ratio; CI = Confidence interval.

Hosmer-Lemeshow goodness-of-fit test = 2.86; p = 0.8261.

^★^Significant at p = 0.048 level.

**Fig 4:**
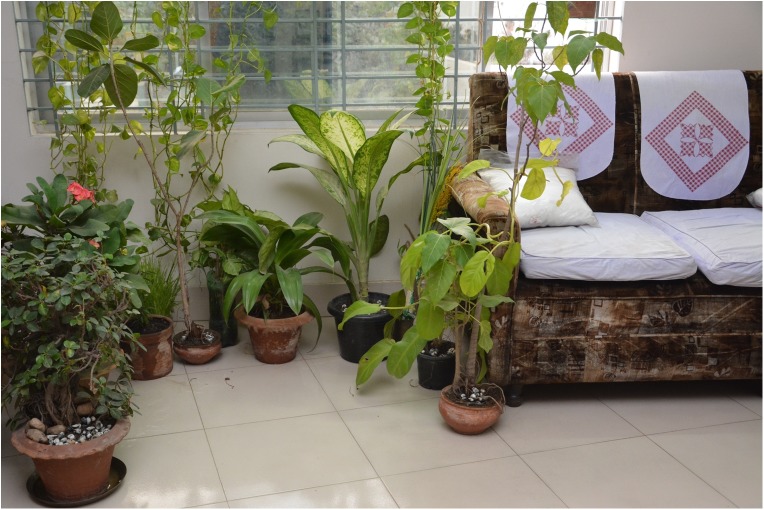
Ornamental potted plants that hold water in containers are common in living rooms of middle and high SES households.

For the age group 12–44, the odds ratio is 4.13, implying that the persons in this age group were 4.13 times more likely to be exposed (i.e., via multiple exposures) to DENV compared to those who were in the group less than 12 years of age. Similarly, persons in the age group above 45 were more likely (OR = 5.9) to be exposed to DENV relative to children (less than 12 years) (Table 5). Persons in household without any indoor potted plants were less likely to be exposed to DENV than those who owned some indoor potted plants (OR = 0.53), suggesting that seroprevalence is positively associated with the presence of indoor potted plants (Fig 4). Mosquito control measures at the personal level were found to be significantly associated with seroprevalence (p = 0.048). While univariate analysis indicated that persons using bed nets were less likely to be exposed to dengue virus than those using sprays or coils, the degrees of difference for these two comparators versus bed nets were deemed to be nominal in multivariate analysis.

A multivariable logistical regression model was used to determine whether the socioeconomic, demographic, behavioral, utensils, ecological (i.e., vegetation or trees nearby) and epidemiological factors (as defined above) were associated with seroconversion. Results from the stepwise logistic regression method, shown in Table 6, revealed that age (χ^2^ = 11.1, df = 1, p-value = 0.001), attendance in public gatherings (χ^2^ = 17.2, df = 1, p-value <0.0001), and types of mosquito control measures used (χ^2^ = 32.5, df = 2, p-value <0.0001) were found to be significantly associated with seroconversion after adjusting all other variables.

**Table 6:** Estimated odds ratios (OR) for the multilevel analysis of the association between seroconversion of dengue and selected explanatory variables, 2012 pre- and post-monsoon serosurveys.

Variable	Estimate (std. error)	OR (95% CI)	p-value
**Intercept**	0.38 (0.50)		0.450
**Attendance in public/mass gatherings**			
Garden/Park	Reference	1	
Others (Schools, Religious Institutions etc.)	-1.66 (0.68)	0.19 (0.05–0.71)	0.0138*
None			
**Type of mosquito control measures**			
Bed nets	Reference	1	
Mosquito coils	2.67 (0.78)	14.55 (3.13–67.7)	
Sprays and others	1.25 (1.29)	3.51 (0.28–43.91)	0.0026*
No Response			

^★^Significant variables at p = 0.05 level

**Fig 5:**
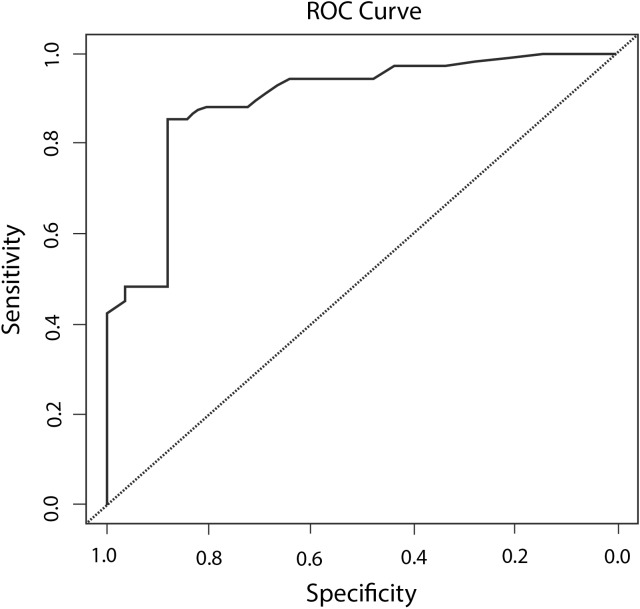
Receiver Operating Characteristic (ROC) curve for the association between seroprevalence and the explanatory variables, 2012 pre-monsoon serosurvey.

As shown in Fig 5, the area under the curve (Receiver Operating Characteristic—ROC) is significantly different from 0.5 since p-value is <0.00001 implying that the logistic regression model used to explain the association between seroprevalence and the explanatory variables classifies the ‘status’ variable significantly better than by chance.

Persons attending schools or religious institutions were less likely (OR = 0.19) to be infected with DENV than individuals who spent time in parks or gardens (Table 6).

The type of mosquito control measures undertaken by household members also significantly affected the patterns in seroconversion; for example, urban residents using mosquito coils were 14.6 times more likely to be recently infected with DENV than bed net users [p-value = 0.0026 (Table 6)]. All other explanatory variables were found not to be significantly associated with dengue seroconversion.

## Discussion

The objectives of this single-year prospective cohort study were to determine dengue seroprevalence, and the rates of seroconversion to DENV during the peak season, and to assess socio-demographic, socio-economic and other risk factors for dengue seropositivity among the population of the city of Dhaka, Bangladesh. All evidence from the serological analysis indicates that a large proportion of the human population of Dhaka was newly exposed to DENV during the period of observation. It also indicates substantial prior background exposure of the general population prior to this period, suggesting endemic dengue virus circulation. Seroprevelance of DENV-specific IgG antibodies was high ranging from 80% among the 1125 pre-monsoon study participants to over 90% for the 600 paired samples collected during post-monsoon time point and the extent of seropositivity increased with age. In addition, the “high” proportion of seroconversion (i.e. 57.7%) signaled that on-going “new infections” were occurring not only during the pre- and post-monsoon periods in 2012 but most likely in past years as well. There were also interesting patterns in the spatial distribution of DENV seroprevalence in Dhaka and it appeared that risk was not uniform across the different City wards. Wards with “very high” degree of seropositivity tended to be those with high density housing (high-rises) and corresponding high human population density and these were located in the older, historically wards of the City; while those with “moderate” degree of seropositivity were located at the edges of the city where housing and human population densities were lower (Fig 2). Similar correlations between human population density and housing conditions and risk of exposure to DENV have been reported elsewhere [35–36]

The higher magnitude of seroconversion among both children and adults during the 2012 pre- and post-monsoon periods indicate intense circulation and transmission of DENV during the study period. The documented seroconversion observed in this study indicates that DENV circulated at a high intensity during the 2012 monsoon season, however, many, if not most, of the infections might have been mild or even asymptomatic. There were no major reports of symptomatic dengue infection, such as dengue haemorrhagic fever, during the observation period. It is also possible that the circulation of DENV in the city may have changed from monotypic to multitypic circulation in recent years and such a shift likely had an important bearing on the epidemiology of dengue in Dhaka.

Overall, the various risk factors and their influence in the city of Dhaka were most likely dependent upon exposure rates resulting from cumulative infections over time for seroprevalence (as measured by persistent IgG), and from a high level of infection in a relatively short period of time for seroconversion. The positive linear relationship between presence of DENV antibodies and age is indicative of cumulative DENV exposure in Dhaka. Many other studies have reported that DENV antibody prevalence increases with age [2, 37–43] and on-going virus transmission, over many years, is often cited as the reason for this pattern [38].

The high seroprevalence in Dhaka should also be regarded as an indicator of significant unreported dengue virus-associated illness. This might represent a major risk factor for secondary DENV infections and more severe diseases, such as dengue haemorrhagic fever, if a new dengue serotype to which there has been limited prior exposure should suddenly be introduced. For instance, in a study of children at public schools in Rio de Janeiro, Brazil, Chunha et al. [44, 45] observed secondary infections among 61% of positive cases; noticeably, 75% of them were in patients under 15 years of age.

**Fig 6:**
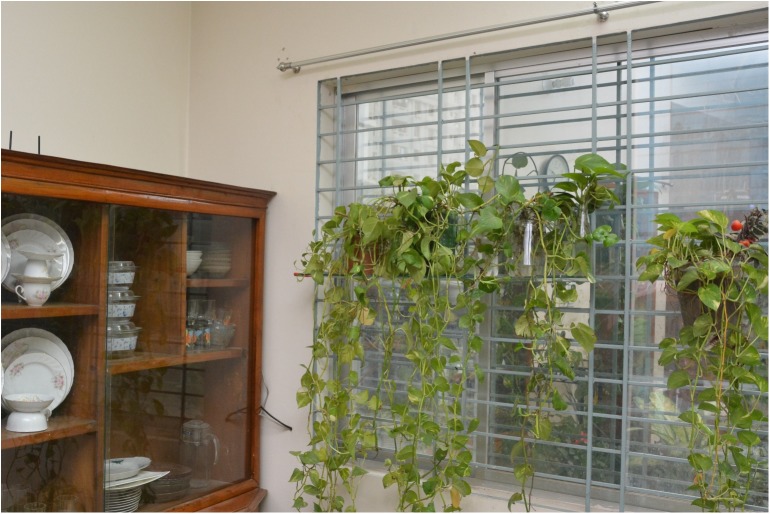
*Epipremnum aureum* plants grown in water in glass and plastic containers are often used to decorate living rooms.

Among children, the burden of exposure is disproportionately higher especially for children >6 years of age. Children in Dhaka may be more vulnerable to dengue infection due to their exposure to infected mosquitoes as they spend most of their time in crowded places (e.g. parks, recreational areas, etc.). It is evident that as soon as children begin to attend elementary school at age 6, the IgG seroprevalence rate increases. Similar associations between rates of DENV seropositivity age of children have been reported in studies conducted in several cities in Asian and Latin American [10, 46–49]. However, the relative vulnerability of children compared to adults is still a subject of considerable debate [50–52], and needs further investigation. It is well established that household ecology, defined as the distribution and abundance of *Ae*. *aegypti* larval development sites, are important risk factors for dengue infection [53–54]. In our univariate and multivariate analyses, the presence of indoor potted plants and the types of mosquito control measures undertaken by household members were the only household level risk factors significantly associated with seroprevalence. These are indicative of the important role that household ecology, especially the clean water-use patterns, and personal bite prevention strategies can have on patterns of DENV exposure in Dhaka. In the case of Bangladesh, with changed social values and expectation for better materialistic life-style in recent decades, keeping potted ornamental plants indoors has become the norm. Housing *Epipremnum aureum* plants (referred to locally as “*money plants”* which grow effectively in small ornamental containers with little water or sunlight) inside homes (Fig 6) for the purpose of decorating living areas symbolizes higher social status [55]. This trend has become more prevalent among the middle and upper-middle socio-economic class, which was reflected in the high proportion of dengue cases among this demographic cohorts during the 2000 epidemic [19].

There have been nominal studies on household level risk factors worldwide. In a study in southern Vietnam, Thai et al. [56] found a positive association between some peri-domestic risk factors including discarded cans and pit latrines and a high prevalence of DENV antibodies. Similar to some of our findings, the pattern of DENV transmission occurring mainly (but not exclusively) at home was observed in studies conducted in Singapore [40], Brazil [41] and Venezuela [11]. Braga et al. [39] observed that household members who spent more time within homes, such as domestic workers and housewives, were at a higher risk of acquiring recent dengue infection.

In the city of Dhaka, Dhar-Chowdhury et al. [30] calculated the Breteau Index (BI), defined as the number of positive (infested) containers per 100 houses inspected, to be quite high ranging from 52.0 to 63.4. In contrast, a study in central Havana, Cuba, reported BI values from 0.11 to 1.32 [57] and these differences likely reflect the relative inefficiency of mosquito control measures undertaken by the public in Dhaka. A recent survey of 300 residents of local urban communities by some of the present authors [58] revealed that although the residents felt that institutional actions could control *Ae*. *aegypti* mosquitoes and mitigate dengue by the use of insecticides (in the form of space sprays), by and large such measures were lacking. Consequently, residents had to implement their own household level mosquito control measures in order to attempt to minimize exposure to vector mosquitoes. The most common types of mosquito control measures employed by residents of Dhaka were use of bed nets, burning of mosquito coils and the use of insecticidal sprays. Consistent with other studies, we observed a significant association of both DENV seroprevalence and seroconversion with use of some personal mosquito control measures [11,59–60]. In our evaluation of the effect of personal mosquito control measures on the risk of dengue infection, bed nets appeared to be associated with a lower likelihood of infection than the use of mosquito coils or insecticide sprays, which is consistent with findings reported by others [61]. This pattern might reflect the overall inefficiency of some of these devices (e.g., mosquito coils or sprays) to reduce vector populations, or using them at inappropriate times (i.e., at night rather than in the daylight hours when *Ae*. *aegypti* are most active). Finally, random error due to small sample size (N = 56) in the some of the analysis (i.e., seroconversions) could not be excluded. Despite the limited observed benefit in our study, use of treated bed nets and mosquito control measures like burning mosquito coils are recommended by the WHO for persons who sleep during the daylight hours and to reduce day-time mosquito biting activity, respectively [62]. Given the lack of municipal level vector control, public health officials in Dhaka should consider reinforcing and educating residents about the proper use and potential benefits of household level vector control measures.

In our study, educational attainment by household heads and the income status of the households were not closely associated with dengue seroprevalence. This finding conforms to the results of a Brazilian study by Vasconcelos et al. [52]. However, numerous studies report that poverty in general (such as, at the local community level) and the low socio-economic status (SES) of residential zone are important risk factors [11,42,63], an incorporation of SES of residential zones, along with individual’s socioeconomic characteristics in future risk factor studies in Bangladesh will help to understand the context of the country better. In a comparative study between Texas, USA and Matamoros, Mexico, Brunkard et al. [42] inferred that poverty is an effective proxy indicator for numerous risk factors. They reported that the protective effect of air conditioning in the more developed, well-off areas was profound in lower risk exposure. In Taiwan, Ko [64] recorded that patients who resided near markets and/or open sewage or ditches had a risk of contracting infectious diseases 1.8 times higher than those who did not live in such conditions. As several analysts already registered [65–69], low socioeconomic residential area risk factor may be attributed to poor settlement and housing structures, high population density, presence of interdomiciliary potential mosquito breeding sites such as, potholes, discarded bottles and cans, vehicle tires in such urban zones. Further multi-scale investigations encompassing both individual and neighborhood level socioeconomic status as risk factors are necessary to fill in this gap.

Reflected in the univariate and multivariate analyses, attendance in public gatherings and the types of mosquito control measures undertaken by the household members were significantly associated with seroconversion. This implies that recent dengue infection takes place in residences located in areas with high mosquito densities (reflected in the need to use mosquito control measures) as well as in outdoor locations where *Ae*. *aegypti* mosquitoes have access to people. Many people would spend much of the daylight hours, when vectors are most active, in these locations. In a study of two major neighborhoods in Rio de Janeiro, Brazil, Honorio et al. [70] observed that recent dengue infections occurred primarily in locations out of residences, either in other premises (such as schools) or out-of-doors. They further argued that investigation on human population movement patterns could bring insights on dengue transmission dynamics and main places of transmission. The seroconversion rate of DENV infection during the post-monsoon period in Dhaka was much higher (57.7%) than recently estimated for regions with stable transmission in South East Asia, such as Southern Vietnam (11.7 cases/100 person-years) [56], Northern Thailand (8.5 cases/100 person-years) [60]; and the Americas, such as Nicaragua (6.0–12.0 cases/100 person-years) [71], and higher than Brazilian Amazonia (3.67 cases/100 person-years) [72]. However, the possibility of a “silent” epidemic during the 2012 pre- and post-monsoon study period may have contributed to an increased force of infection resulting in a high rate of seroconversion among both children and adults. Previous studies have shown that the introduction of a “new” dengue serotype during an outbreak can result in seroincidences of greater than 50% [73–74]. It is possible that a heightened transmission intensity in Dhaka in 2012 gave rise to the observed rates of new infections among seronegative individuals. However, due to the relatively small number of individuals studied for seroconversion and possible selection bias, seasonal exposure rates may also be due at least in part to an ongoing high endemic incidence of transmission. Further studies analyzing differences in seroconversion rates for various city zones may shed further light on the risk factors involved in viral transmission.

In contrast to seroprevalence, age was not found to be a significant factor in seroconversion. The seroconversion rate for children (under 12 years of age) and for persons aged 12 years and more was approximately 50%. The high risk factor for seroconversion among the children in the context of the city of Dhaka, as stated earlier, can partially be attributed to time spent by children in communal settings (schools and post-school settings). In Dhaka, the density of child population (age group: 6–11 years) is higher in primary schools, playgrounds, and post-school nurseries and activity in these areas, during the daylight hours, is likely to result in exposure to *Ae*. *aegypti* mosquitoes. Such exposure among children has also been recorded in seroconversion among school children during epidemics in Puerto Rico [43], Peru [72], the Dominican Republic [48], Brazil [75], and Thailand [60].

Based on existing literature on dengue in Bangladesh collected after 2000 epidemic, it is inferred that most dengue cases in Dhaka are caused by DENV-3 [19–20,29], with co-circulation of DENV-2 and DENV-4 [20]. However, comprehensive population-based study of serotype has yet not been pursued. Although serotype identification was beyond the scope of the present research, in a separate study under our research project, we have performed PCR of 47 acute dengue cases serum samples from hospitalized patients. It is intriguing that our results identified DENV-1 serotype among 3 PCR positive samples and these samples shared 99% homology with DENV isolated from Delhi, India [76]. The identification of the dengue 1 serotype may be possibly indicative of an outbreak or increased circulation / introduction of this particular serotype in 2012 or within a similar time period. This may have contributed to the high seroconversion rates observed during the pre–post monsoon seasons.

In conclusion, to our knowledge, this is the first population-based study of DENV infection among the population of the city of Dhaka, Bangladesh, revealing a high DENV seroprevalence with a considerable degree of seroconversion over the monsoon season in 2012. It is possible that the high magnitude of seroprevalence and seroconversion are significantly contributing to under-recognized mild and severe disease in Dhaka–a major concern for the South Asian regional, national and local public health authorities. In addition, the findings of our study have indicated that home-based interventions such as, removing indoor potted plants and increased bed net use, in addition to vector control measures in public parks, would reduce exposure to dengue infections among the residents of the city of Dhaka. Nonetheless, in the absence of an active surveillance and effective public health measures, understanding and preventing spread of DENV among the population would remain poor. Formulation of an active DENV surveillance system is urgently warranted, particularly to estimate the impact of DENV dengue virus and DF and DHF disease burden in the city of Dhaka and other urban areas of the country.

Although, the risk for JEV and WNV in Dhaka is very low based on serological results from the current study, it is possible that JEV may still circulate at low levels in the city since the virus was previously detected in rural cases by hospital-based surveillance in four sites of Bangladesh [77]. Other mosquito-borne viruses such as Chikungunya (CHIKV) [78] and Zika virus may also contribute to illness in Dhaka and other regions within Bangladesh.

Hyper- endemic settings of this South Asian region for circulation and transmission of CHIKV has also been reaffirmed by a recent study in India which reported 44% seropositivity to CHIKV [79]. The increasing likelihood of co-transmission of arbovirus diseases, the emerging Zika virus risk [80], the global spread of *Ae*. *aegpyti* mosquitoes, and effects of increasing international human mobility should set the agenda for undertaking similar research studies characterizing their prevalence in Dhaka and the diagnostic and public health challenges presented by their potential incursions.

### Limitations

This study was subject to several limitations. Firstly, the participation rate was low during the follow-up study. Due to a very high rate of change of residence and relocation spatially among Dhaka city dwellers, we collected only 600 paired sera samples during the post-monsoon survey, causing us to limit the sample size to only 50% of the initial target. A larger sample size in future studies will provide a higher degree of confidence when interpreting serological outcomes. The possibility of information bias in our study overall is nominal. This is because all potential *Aedes* breeding sites in household premises were inspected with their complete (total) counts; for example, field entomologists counted all potted plants, uncovered water tanks, number of trees nearby and thus, there was no possibility of information bias. However, there still could be some degree of information bias in our analysis due to the sampling error in the population estimates. As well, we were unable to determine the infecting DENV serotype responsible for the observed dengue seroconversion and future studies are warranted to identify dengue serotypes and strains currently circulating in the city. Lastly, because this is the first population-based DENV seroprevalence study in Bangladesh, we are unable to compare our findings with other seroprevalence data in the country and we may have underestimated the seroincidence rate, because we relied on collecting specimens from our baseline study population only.

## Supporting information

S1 Checklist (PDF)

S1 Table Number of counts (sample) and percentage of seropositive (IgG) cases by age groups (all ages), 2012 pre-monsoon serosurvey. (DOC)

S2 Table Number of counts (sample) and cumulative percentage of seropositive (IgG) cases among the children (≤12 years) by year, 2012 pre-monsoon serosurvey. (DOC)
