# Supplementary material for: Dengue seroprevalence, seroconversion and risk factors in Dhaka, Bangladesh
Source: PLoS Negl Trop Dis. 2017 Mar 23;11(3):e0005475. doi: 10.1371/journal.pntd.0005475 (PMC5380355; doi:10.1371/journal.pntd.0005475)
Supplement: S1 Table — (DOC) [file pntd.0005475.s002.doc]

**S1 Table:** **Number of counts (sample) and percentage of seropositive (IgG) cases by age groups (all ages), 2012 pre-monsoon serosurvey.**

| Age Groups | Positive (Count) | % Seropositve (IgG) | Negative (Count) | Total (Count) |
| --- | --- | --- | --- | --- |
| <12 | 67 | 54.9 | 55 | 122 |
| 12-22 | 189 | 71.6 | 75 | 264 |
| 23-33 | 263 | 83.2 | 53 | 316 |
| 34-44 | 205 | 91.9 | 18 | 223 |
| 45-55 | 114 | 85.1 | 20 | 134 |
| 56-66 | 51 | 91.1 | 5 | 56 |
| 67 ≥ | 11 | 100 | 0 | 11 |
