# Supplementary material for: Dengue seroprevalence, seroconversion and risk factors in Dhaka, Bangladesh
Source: PLoS Negl Trop Dis. 2017 Mar 23;11(3):e0005475. doi: 10.1371/journal.pntd.0005475 (PMC5380355; doi:10.1371/journal.pntd.0005475)
Supplement: S2 Table — (DOC) [file pntd.0005475.s003.doc]

**S2 Table: Number of counts (sample) and cumulative percentage of seropositive (IgG) cases among the children (≤12 years) by year, 2012 pre-monsoon serosurvey.**

| Age (Children) | Positive (Count) | Cumulative % to all children age groups | Negative (Count) | % to all children  age groups |
| --- | --- | --- | --- | --- |
| 1 | 1 | 1.5 | 1 | 1.5 |
| 2 | 5 | 9 | 6 | 7.5 |
| 3 | 1 | 10.5 | 4 | 1.5 |
| 4 | 8 | 22.4 | 6 | 11.9 |
| 5 | 4 | 28.4 | 9 | 6 |
| 6 | 3 | 32.9 | 3 | 4.5 |
| 7 | 11 | 49.3 | 9 | 16.4 |
| 8 | 6 | 58.3 | 4 | 9 |
| 9 | 8 | 70.2 | 5 | 11.9 |
| 10 | 7 | 80.6 | 6 | 10.4 |
| 11 | 13 | 100 | 2 | 19.4 |
